# Tumeur desmoïde de la glande Bartholin: à propos d'un cas

**DOI:** 10.11604/pamj.2015.22.49.5259

**Published:** 2015-09-18

**Authors:** Ihssane Hakimi, Farid Kassidi, Hafsa Chahdi, Youssef Benabdjlil, Jaouad Kouach, Driss Moussaoui, Mohamed Dehayni

**Affiliations:** 1 Service de Gynécologie Obstétrique, Hôpital Militaire d'Instruction Mohamed V, Rabat, Maroc; 2 Service d'Anatomie Pathologique, Hôpital Militaire d'Instruction Mohamed V, Rabat, Maroc

**Keywords:** Fibrome desmoïde, tumeur vulvaire, pathologie de la glande Bartholin, desmoid fibroma, vulval tumor, Bartholin gland pathology

## Abstract

Les tumeurs desmoides sont les tumeurs rares, bénignes mais fréquemment agressives d'origine mésenchymateuse, ils sont extrêmement rares en localisation Vulvaire. Nous rapportons le cas d'une patiente âgée de 32 ans présentant un fibrome desmoïde de la glande de Bartholin prise pour un simple kyste au début. Le traitement consiste en une excision chirurgicale de la lésion et nous discutons la possibilité de traitement adjuvent pour éviter une éventuelle récidive post-opératoire.

## Introduction

Les tumeurs Desmoides sont premièrement décrite par Mac Farlane en 1832. Le terme « desmoid » a été attribué à ces tumeurs par Muller en 1838 pour indiquer leur aspect macroscopique caractéristique et pour le donner une consistance rugueuse. En 1923, Nichols et al a montré l´association du polypose familial avec les tumeurs desmoides. Les tumeurs Desmoides sont les tumeurs rares, bénignes mais fréquemment agressives d´origine mésenchymateuse, formant un groupe d´entités pathologiques hétérogène résultant de la prolifération des fibroblastes bien-différenciés. Aujourd´hui, il est bien connu que la plupart des tumeurs desmoid se produisent sporadiquement, et chez ces patients ils sont trouvés généralement dans la cavité abdominale ou la paroi abdominale. D´autres emplacements possibles des tumeurs desmoides sont le tronc ou les extrémités [1]. Dans notre cas, la tumeur est de localisation vulvaire au niveau de la glande Bartholin. La gestion clinique des patients avec les tumeurs desmoides est difficile et controversée. Beaucoup de questions demeurent sans réponse concernant le dépistage précoce, le rôle de la chirurgie (indication, rôle, synchronisation et ampleur), et la place du traitement conservateur [1].

## Patient et observation

**Figure 1:**
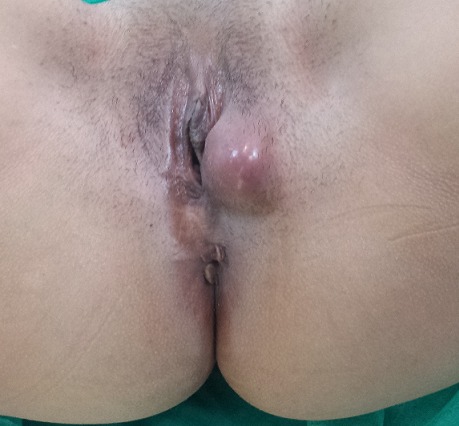
Tuméfaction de la glande de Bartholin

**Figure 2:**
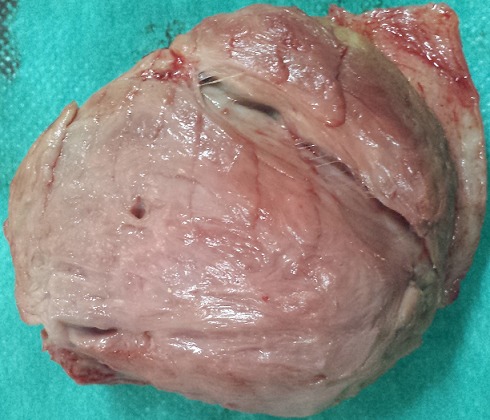
Piece opératoire de la tumeur après chirurgie

**Figure 3:**
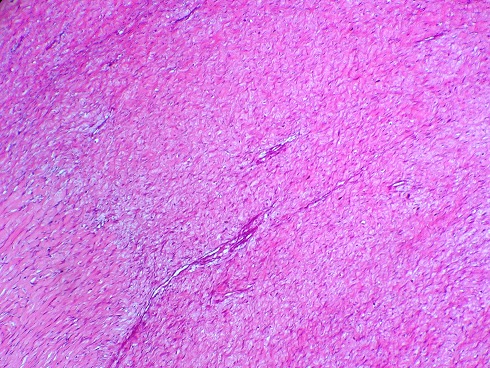
Prolifération de cellules fusiformes régulières, disposées en faisceaux, et entourées de nombreuses fibres de collagène

Il s'agit de Mme R.K âgée de 32 ans multipare avec un cycle menstruel régulier, ayant accouchée trois fois par voie basse et sans antécédents pathologique notable, s'est présentée en consultation pour une tuméfaction vulvaire apparue depuis 4 mois augmentant progressivement de volume. L'examen gynécologique est sans particularités en dehors de la voussure vulvaire au niveau de la glande de Bartholin donnant l'aspect d'un simple kyste de cette glande sauf qu'elle est de consistance ferme (Figure 1). Le tout évoluant dans un contexte d'apyrexie et de conservation de l’état général. L’échographie pelvienne est strictement normale, l'utérus de taille normale d’écho structure homogène, les annexes sont normaux, sans épanchement dans le douglas. Une excision chirurgicale a été proposée à la patiente puis réalisée permettant d'enlevé la masse ferme fibreuse de dissection relativement difficile avec absence de plan de clivage. L'aspect macroscopique de la tumeur était celui d'une tumeur fibreuse (Figure 2). L'histologie a posé le diagnostic de fibromatose (ou tumeur) desmoïde (Figure 3).

## Discussion

Les fibromes desmoïdes sont un groupe de tumeurs fibreuses profondes, cliniquement hétérogènes, regroupées sous le vocable des « fibromatoses desmoïdes ». Elles sont classées selon leur comportement biologique en trois groups: sporadique, associé à une polypose adénomateuse familiale, et la forme familiale ou multicentrique [2]. La forme infantile est parfois décrite comme un quatrième groupe. Parallèlement, ces tumeurs ont également été classifiées selon leur localisation anatomique en extra-abdominales, pariétales abdominales ou intra-abdominales. Le pic d'incidence est situé entre 20 et 40 ans. Ces tumeurs surviennent deux fois plus souvent chez la femme. Si leur étiologie reste actuellement largement inconnue, plusieurs hypothèses ont été proposées. Le rôle de l'imprégnation hormonale a été évoqué par plusieurs auteurs suggérant que les tumeurs desmoïdes soient estrogéno-dépendantes.

Le rôle d'un traumatisme, y compris celui causé par une chirurgie antérieure, a également été évoqué [3]. Néanmoins, leur existence en tant que lésions réactionnelles semble remise en cause actuellement, étant donné le rôle prépondérant que semble jouer le facteur génétique [2]. En raison de l'absence de potentiel métastatique et de leurs caractéristiques microscopiques dépourvues d'atypies nucléaires, ces tumeurs sont considérées comme bénignes [2, 4]. Cependant, elles furent encore classées récemment parmi les fibrosarcomes de bas grade en raison de leurs capacités d'invasion locale et de récidive. En effet, s'il n'existe pas actuellement de série clinique suffisamment large permettant de quantifier le risque avec précision, les taux de récidives varient entre 25 et 50% [4, 5]. La symptomatologie des fibromes desmoïdes n'est pas très spécifique. Leur localisation vulvaire est exceptionnelle. Nous n'avons retrouvé aucun cas décrits dans la littérature.

Le diagnostic est histologique. Les tumeurs desmoides partagent presque toutes les mêmes caractéristiques histologiques [2]. Les tumeurs Desmoides sont des proliférations des cellules mésenchymateuses qui peuvent surgir en n´importe quelle structure musculo-aponévrotique. La nature précise de la cellule impliquée est peu claire, mais les cellules montrent la morphologie de fibroblaste et peuvent être d´origine de myofibroblaste. Au microscope, des tumeurs desmoides sont mal entourées, infiltrant le tissu environnant, et manquant d´une vraie capsule. Elles se composent de collagène abondant entourant les paquets mal entourés de cellules ovales, minces, fusiformes d´aspect uniforme [1]. Le traitement de choix est la chirurgie. Ces tumeurs doivent être excisées « en bloc » avec une marge suffisante [6]. Cependant, la réalisation de marges de sécurité, ne permet souvent pas d’éviter une récidive, et reste donc très controversée [5]. Dans notre observation elle a été réalisée un excision chirurgicale de la masse sans marges de sécurité vu que le diagnostic n’était pas connu. Des contrôles cliniques fréquents sont à recommander afin de détecter précocement toute récidive, bien qu'aucun consensus thérapeutique ne soit défini dans ces cas. Une résection chirurgicale n’étant pas toujours réalisable, les traitements médicaux s'imposent alors comme ultime recours [7].

Par ailleurs, le rôle des traitements complémentaires à la chirurgie première reste controversé. La majorité des données fournies par la littérature se réfèrent à des études rétrospectives ou à des cas anecdotiques. La radiothérapie a ainsi été utilisée comme traitement néo-adjuvant, adjuvant, et parfois en remplacement de la chirurgie. L'effet de diverses combinaisons d'agents chimio thérapeutiques, dont l'association Vinblastine/Méthotrexate fût une des plus utilisées, n'a jamais été démontré sur un grand nombre de patients. Enfin, le traitement hormonal par Tamoxifène a montré des effets favorables, sans doute par son interaction avec les récepteurs aux estrogènes qui seraient exprimés en grand nombre par les cellules des tumeurs desmoïdes. Ces données restent elles aussi limitées à un nombre restreint de patients [5, 7, 8]. Dans notre cas la patiente n'a reçu aucun traitement adjuvent juste une surveillance clinique rapprochée depuis 3mois ne montrant aucun signe de récidive locale.

## Conclusion

Les fibromes desmoïdes vulvaire représentent une localisation rare au sein d'une entité clinique connue pour son caractère localement invasif. Ceci rend le traitement chirurgical définitif de ces lésions difficile et le taux de récidive est élevé.
